# Biomarkers Predictive of Immunotherapy Efficacy in Head and Neck Squamous Cell Carcinoma: Current Insights and Future Perspectives

**DOI:** 10.1002/wjo2.70105

**Published:** 2026-07-05

**Authors:** Xiao‐Tong Yan, Zhao‐Hong An, Chang‐Ming An

**Affiliations:** ^1^ Department of Head & Neck Surgery, National Cancer Center/National Clinical Research Center for Cancer/Cancer Hospital Chinese Academy of Medical Sciences and Peking Union Medical College Beijing China

**Keywords:** biomarkers, head and neck squamous cell carcinoma, immunotherapy, tumor microenvironment

## Abstract

With the approval of immunotherapies by regulatory bodies, head and neck squamous cell carcinoma (HNSCC) treatment has been revolutionized, and anti‐programmed cell death‐1 (anti‐PD‐1) agents are now the standard for platinum‐resistant recurrent/metastatic HNSCC. However, programmed death ligand 1 (PD‐L1) expression does not always align with treatment efficacy, and given the risk of immune‐related toxicities and high costs, identifying predictive biomarkers is crucial. This review comprehensively examines the current evidence for diverse biomarkers predictive of HNSCC response to anti‐PD‐1/PD‐L1 therapy, including but not limited to PD‐L1 expression, tumor mutational burden (TMB), features of the tumor microenvironment (TME), and circulating biomarkers. This integrative approach aims to overcome current limitations, comprehensively evaluate immunotherapy response potential, enhance prognostic assessment, and ultimately guide personalized treatment strategies in clinical practice for HNSCC patients.

## 1 Introduction

Head and neck squamous cell carcinoma (HNSCC), which ranks seventh among global malignancies, represents approximately 90% of head and neck cancers. It predominantly originates in the mucosal linings of the oropharynx, hypopharynx, and laryngeal regions [1]. Global estimates indicate that over 600,000 new cases are diagnosed annually, with fatalities exceeding 300,000 each year [2]. Significant geographic variation in incidence correlates strongly with lifestyle exposures—including tobacco use, alcohol intake, and betel quid consumption—alongside genetic susceptibility [3]. Critically, human papillomavirus (HPV) infection has emerged as a major etiological driver; its oncoproteins E6 and E7 disrupt cellular tumor suppressor pathways, accelerating carcinogenesis [3, 4].

Immunotherapeutic strategies have transformed oncology, with programmed death‐1 (PD‐1)/PD‐L1 inhibitors becoming cornerstone interventions. Pembrolizumab and nivolumab gained regulatory approval for the treatment of platinum‐refractory HNSCC in 2016. By 2019, pembrolizumab had advanced to first‐line status for recurrent/metastatic head and neck squamous cell carcinoma (R/M HNSCC) and was administered alone or alongside cytotoxic agents [5]. Neoadjuvant applications show particular potential. The KEYNOTE‐689 study revealed a decrease in tumor stage in 19% of HNSCC patients presurgery, coupled with low immune toxicity [6]. CheckMate‐358 further established the safety of neoadjuvant nivolumab, observing pathological responses irrespective of HPV status in locally advanced cases [7]. In addition, our team's 2020 phase II trial (NCC2020C 471) evaluated toripalimab combined with chemotherapy in locally advanced HNSCC. Within a cohort of 22 participants who received pembrolizumab, cisplatin, or paclitaxel preoperatively (median follow‐up: 9.5 months), the outcomes included a 78.3% pathological response rate, 36.4% complete pathological remission, and 90.9% laryngeal preservation. Only one individual (4.6%) experienced locoregional recurrence. Meanwhile, the combination of CTLA‐4 inhibitors with PD‐1 blockade has emerged as a pivotal strategy, which not only synergistically enhances anti‐tumor responses but also counteracts the expansion of immunosuppressive Treg cells potentially induced by PD‐1 monotherapy in recurrent/metastatic settings [8, 9]. Furthermore, novel PD‐1/CTLA‐4 bispecific antibodies, such as cadonilimab, combined with chemotherapy have demonstrated high pathological response rates in clinical trials [10].

Nevertheless, reliable predictors and assessment tools for immunotherapeutic efficacy remain limited. While a PD‐L1 combined positive score (CPS) ≥ 1 correlates with improved responses in KEYNOTE 055 and CheckMate 14, individuals scoring a CPS < 1 still derive substantial clinical benefit. These findings underscore the inadequacy of PD‐L1 as a standalone predictive or evaluative biomarker [11]. Additional candidates—including immune cell infiltration patterns, tumor mutational burden, and transcriptomic signatures—are under investigation but lack validation in HNSCC contexts.

**Figure 1:**
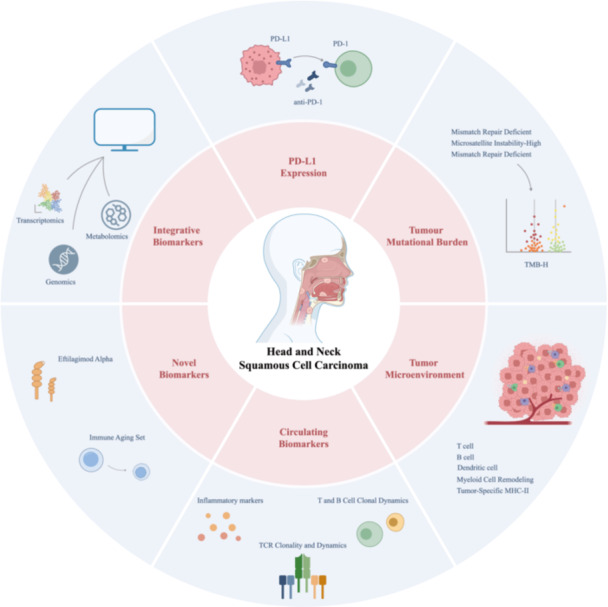
Classification of predictive biomarkers for immunotherapy efficacy in head and neck squamous cell carcinoma. This schematic delineates key biomarker categories under investigation for predicting responses to anti‐PD‐1/PD‐L1 immunotherapy in HNSCC. The framework integrates (1) PD‐L1 expression, (2) tumor mutational burden, (3) tumor microenvironment (TME), (4) circulating biomarkers, (5) novel biomarkers, and (6) integrative biomarkers.

This analysis consolidates present research on biomarkers linked to anti‐PD‐1/PD‐L1 responsiveness in HNSCC. It further advocates the integration of artificial intelligence and machine learning to construct multidimensional, data‐centric models. These frameworks may provide deeper mechanistic insights and refine prognostic accuracy in clinical oncology (Figure 1).

## 2 Expression of PD‐L1

The transmembrane immunoregulatory protein PD‐L1 (CD274) serves as a critical modulator of T‐cell activity. Its interaction with the PD‐1 receptor governs key immunological checkpoint functions [12]. Under physiological conditions, PD‐L1 is constitutively expressed on antigen‐presenting cells, myeloid lineages, and activated T lymphocytes [13]. Emerging evidence indicates that numerous solid neoplasms exhibit dysregulated PD‐L1 expression. This ligand–receptor dyad represents a therapeutic target since malignant cells frequently exploit this pathway for immune evasion [14]. Currently, tumor PD‐L1 quantification remains the foremost biomarker for predicting immunotherapy responsiveness. Elevated PD‐L1 correlates with adverse prognoses across 32 human malignancies, including head and neck carcinomas [15]. Within HNSCC cohorts, PD‐L1‐positive lesions typically demonstrate superior anti‐PD‐L1 treatment outcomes.

### 2.1 Survival Benefits in PD‐L1‐Positive HNSCC Patients

Numerous clinical trials assessing the efficacy of immune checkpoint inhibitors (ICIs) in R/M HNSCC have shown similar conclusions. Multiple clinical investigations of ICIs in recurrent/metastatic disease have reported consistent findings [16, 17]. Phase III data have demonstrated that pembrolizumab significantly prolongs survival in PD‐L1‐positive HNSCC patients. The KEYNOTE‐048 trial (NCT02358031) further established that enhanced efficacy correlated with increased PD‐L1 expression, as evidenced by a survival advantage in R/M HNSCC cohorts receiving pembrolizumab [18].

### 2.2 Diagnostic Ambiguity Regarding PD‐L1 Expression

Contradictory evidence exists regarding the prognostic utility of PD‐L1. CHECKMATE‐141 revealed no significant correlation between PD‐L1 status and survival outcomes during nivolumab therapy for R/M HNSCC [19]. PD‐L1 demonstrates variable predictive utility across HNSCC immunotherapy contexts. Lee et al.'s 2021 Phase III trial revealed potential progression‐free survival benefits in locally advanced patients with elevated PD‐L1 expression receiving chemoradiation plus nivolumab, although wide confidence intervals have limited clinical interpretability [20]. Similarly, Sacco et al. reported robust antitumor activity (ORR 45%; median OS 18.4 months) with cetuximab plus pembrolizumab in recurrent/metastatic disease but reported no significant association between PD‐L1 expression levels and therapeutic outcomes. Meanwhile, a study focusing on oral squamous cell carcinoma (OSCC) patients receiving postoperative adjuvant radiotherapy or chemoradiotherapy revealed that among those undergoing radiotherapy‐based adjuvant treatment, high PD‐L1 expression might not predict therapeutic benefit. Instead, it could identify a subgroup characterized by resistance to current standard therapies and poor prognosis. In these patients, the prognostic and predictive value of PD‐L1 is highly context‐dependent, serving more to guide the selection of treatment modalities than merely predicting the efficacy of immunotherapy alone [21].

### 2.3 The Complexity of PD‐L1 Assessment

The inconsistencies in the above‐described study results may arise from various sources. There is an absence of consistency in the methodologies employed to evaluate PD‐L1 expression across studies, with no consistent threshold for defining PD‐L1 positivity, resulting in diversity in detection outcomes [22]. The principal reagents employed for PD‐L1 immunohistochemistry detection are 22C3 pharmDx and the SP263 assay. Research indicates that the SP263 assay generally yields elevated PD‐L1 scores for both the tumor proportion score (TPS) and the CPS in comparison to the 22C3 pharmDx. Significant discrepancies between the two detection approaches have been reported when tumors are categorized as PD‐L1 positive or negative, especially at the key criteria of a CPS ≥ 20 and a TPS ≥ 50% [23]. Moreover, PD‐L1 expression is modulated by many signaling pathways, cytokines, and inflammatory mediators, including MAPK, PI3K/Akt/mTOR, and NF‐κB [24, 25, 26, 27], which enhance its dynamic characteristics. Tumor expression fluctuates during initiation and progression in response to tumor formation and recurrence. Moreover, substantial disparities exist in PD‐L1 expression both intratumorally and between original and metastatic lesions [28]. The temporal variability and spatial heterogeneity of PD‐L1 expression result in inconsistencies in detection outcomes.

The tumor microenvironment (TME) comprises several cytokines and inflammatory mediators that modulate PD‐L1 expression. Recent studies have shown that interferon‐gamma (IFN‐γ) promotes PD‐L1 expression in oral squamous cell carcinoma (OSCC) cells by upregulating downstream target protein kinase D isoform 2 (PKD2) in a time‐ and dose‐dependent manner, and this effect is IFN‐γ dependent [29]. Furthermore, studies have shown that CMYM6, a Type III transmembrane protein, increases PD‐L1 synthesis in HNSCC cells, decreases CD8^+^ and CD4^+^ T‐cell infiltration [30], and protects PD‐L1 from ubiquitination in tumor cells, thus prolonging the half‐life of the protein. PD‐L1 expression has dynamic effects on multiple regulatory variables [31].

PD‐L1 distribution exhibits significant spatial heterogeneity across primary/metastatic sites and intratumoral regions. Immunohistochemical analyses revealed dual subcellular localization, membranous and cytoplasmic PD‐L1 expression [32], indicating two distinct spatial configurations: diffuse infiltration throughout neoplastic tissue and peripheral concentration bordering tumor margins. The highest expression density occurs at invasion fronts, particularly within the tumor‐adjacent stroma and tumor‐associated macrophages (TAMs) [33]. This spatial inconsistency introduces prognostic variability when PD‐L1 serves as a predictive biomarker for HNSCC immunotherapy.

### 2.4 Advancing PD‐L1 Detection Sensitivity

In the study by Yuri et al., the assessment of PD‐L1 expression using the Clone 73‐10 Antibody has demonstrated considerable clinical relevance [34]. The study reports PD‐L1 positivity (defined as TC ≥ 1%) in 79% of HNSCC cases, which correlates with unfavorable patient prognosis. This assay exhibits enhanced sensitivity compared to other clones due to its specificity for the intracellular domain of PD‐L1, enabling more consistent detection despite tumor heterogeneity and potentially expanding the population eligible for ICIs. Mechanistically, it is noteworthy that while PD‐L1 is significantly upregulated, its receptor PD‐1 and CTLA‐4 do not show concurrent upregulation, suggesting PD‐L1 may represent a more effective therapeutic target. Current evidence remains limited by single‐center study designs and a lack of post‐treatment samples. Future large‐scale studies are warranted to further validate the clinical utility of the 73‐10 assay across different scoring systems and treatment contexts [34]. Further contributing to this field, 124/125I‐CS1001, a novel PD‐L1‐targeting radiotracer, was successfully developed and validated for its specific targeting capability through in vitro experiments in CHO and HeLa cells, as well as in vivo animal models. The results demonstrated that 124I‐CS1001 exhibited higher uptake in CHO‐hPD‐L1 tumor models and prolonged tumor retention compared to the small‐molecule probe 68Ga‐WL12. Notably, at 96 h post‐injection, it showed superior tumor‐to‐background ratios and enhanced imaging quality, supporting its potential as a molecular imaging strategy for predicting immunotherapy efficacy [35].

In summary, while substantial evidence demonstrates a general correlation between tumor PD‐L1 expression levels and response to anti‐PD‐1/PD‐L1 immunotherapy in HNSCC, its predictive utility has notable limitations. On the one hand, some PD‐L1‐negative patients still benefit from ICIs; on the other hand, the lack of standardized detection methods and the inherent spatiotemporal heterogeneity of PD‐L1 expression collectively restrict its reliability as a standalone biomarker for clinical decision‐making. Recent studies suggest that optimized assays such as those employing more sensitive clones like 73‐10, which targets the PD‐L1 intracellular domain, may improve detection consistency, partially mitigate challenges posed by tumor heterogeneity, and better identify potential responders. Further large‐scale studies are needed to validate the clinical utility of these novel assays and advance standardization efforts, thereby enhancing the predictive power of PD‐L1 in HNSCC immunotherapy.

## 3 Tumor Mutational Burden (TMB)

The tumor somatic mutation count per megabase (Mb) of the coding genome is called the TMB. These mutations include single‐nucleotide changes, insertions, and deletions. This can be accomplished with next‐generation sequencing, which includes whole‐exome and whole‐genome sequencing.

TMB‐high cancers produce more neoantigens, which help the immune system recognize and target tumor cells, increasing the success of immunotherapy. These conditions may make ICI‐induced antitumor immune responses more effective at eliminating tumor cells [36]. A study of 45 clinical studies and 103,078 cancer patients revealed that increased TMB (≥ 10 mutations per megabase) may be a negative prognostic factor for nonimmunotherapy patients. Regardless of the cancer type or TMB detection method, immunotherapy patients with higher TMB had better OS, PFS, and ORR [37]. In the landmark KEYNOTE‐158 Phase II study, 805 individuals with unresectable solid malignancies were stratified by TMB status prior to pembrolizumab administration. The cohort exceeding the established high‐TMB threshold (≥ 10 mutations per megabase) demonstrated superior objective response rates (29%; 95% CI 21%–39%) compared with 6% (95% CI 5%–8%) in the lower‐TMB subgroup [38].

TMB demonstrates predictive utility for immunotherapy outcomes in HNSCC, yet the complexity of the TME limits its standalone biomarker reliability. Schumacher et al. (2019) reported that 54.5% of TMB‐high patients failed to achieve objective responses, underscoring inherent limitations when utilized as a solitary predictive metric [39]. Initially, only a minor proportion of nonsynonymous mutations produce neoantigens that are identifiable by T cells. Second, the clonality and specificity of these neoantigens, in conjunction with the genetic characteristics of the tumor, facilitate the development of a targeted and efficacious antitumor response. Third, elements pertaining to the host's immunological milieu affect T‐cell‐mediated tumor eradication, including T‐cell trafficking to the tumor site, the equilibrium between activating and inhibitory cytokines, and the nature of tumor immune checkpoints, among other factors. Numerous elements within the tumor immunological microenvironment influence the effectiveness of ICI therapy [40].

Several lines of research suggest that TMB is a viable biomarker for predicting HNSCC immunotherapy efficacy, specifically for immune checkpoint blockade drugs. The FDA has approved pembrolizumab, an anti‐PD‐1 drug, for treating solid tumors with a TMB ≥ 10 mutations/Mb. However, the development of TMB as a clinical biomarker faces considerable challenges. Mutational burden is measured via different TMB thresholds across studies, reducing comparability. Therefore, the standardization and regulatory refinement of TMB must be improved to expand its biomarker use.

## 4 TME

The TME is an intricate ecosystem comprising malignant cells and associated stromal components. This biological milieu mediates pivotal functions across malignant transformation, disease progression, and therapeutic responsiveness. Cellular constituents include endothelial cells, immune populations, vascular networks, lymphatic structures, and cancer‐associated fibroblasts (CAFs) [41]. Contemporary oncology recognizes immune evasion as a fundamental hallmark of malignancy, with multiple TME pathways now targeted by immunotherapeutic interventions [42].

### 4.1 Immune Characteristics of HNSCC

The limited efficacy of ICIs in most HNSCC patients stems from the profound complexity of the TME and the dynamic interplay among its cellular constituents. This immunosuppressive landscape comprises diverse immune populations, including tumor‐infiltrating lymphocytes (TILs), natural killer (NK) cells, antigen‐presenting cells (dendritic cells, macrophages), and myeloid‐derived suppressor cells (MDSCs). Collectively, these cellular interactions establish an immunological signature with significant prognostic implications and biomarker utility for therapeutic response prediction [43]. HNSCC can be classified into three distinct immune microenvironment types on the basis of the immune cell composition in tumor tissues: inflammatory, immune‐excluded, and immune‐depleted [44]. Tumor immunotherapy is effective solely in cancers exhibiting an immune‐infiltrated phenotype [45]. Noninflammatory tumors exhibit activation of the WNT–β‐catenin pathway, which is characterized by reduced CCL4 expression and defective recruitment of BATF3+ dendritic cells (DCs), as well as MYC activation and additional alterations, resulting in decreased immune infiltration [46].

### 4.2 TME as a Biomarker for the Effectiveness of Immunotherapy

#### 4.2.1 T Cell

Regardless of tumor location, stage, or therapy, the only immune cell type associated with improved survival in HNSCC patients is increased infiltration of CD8^+^ T cells [47, 48, 49]. A study by Hanna GJ et al. demonstrated that each unit increase in the infiltration density of CD3^+^/CD8^+^ T cells within the TME was associated with a 2.2% reduction in the risk of death. This finding suggests that CD3^+^/CD8^+^ T cell infiltration holds promise as a biomarker for predicting immunotherapy response in patients with HNSCC [50]. Even though people with HPV‐negative HNSCC sometimes have lower overall CD8^+^ T‐cell counts, they often have the same favorable results as people with HPV‐positive tumors when the fraction of CD8^+^ T cells is identical [49]. A 2018 study obtained similar results for HPV‐negative OPSCC patients treated comparably to HPV‐positive patients, showing that tissue‐resident CD8^+^ T‐cell counts were equivalent. Evidence such as this points to the importance of tissue‐resident CD8^+^ T cells for antitumor immunity in HNSCC, independent of HPV status [51, 52, 53]. Similarly, a recent study employing flow cytometry and single‐cell transcriptome sequencing analyzed immune infiltration in a large cohort of fresh tissues from diverse anatomical sites. It revealed that tumor‐infiltrating PD‐1^+^CD8^+^T cells, particularly an activated subset co‐expressing effector molecules such as CXCL13, are significantly associated with patient prognosis following immunotherapy in HNSCC. This finding has been further validated in other cancer types, including gastric cancer, melanoma, and non‐small cell lung cancer [54]. Advanced HNSCC patients exhibiting elevated intratumoral CD103^+^CD8^+^ T‐cell infiltration demonstrated an enhanced response to neoadjuvant chemoradiotherapy. Experimental evidence indicates that adoptive transfer of these lymphocytes potentiates anti‐PD‐1 efficacy in tumor suppression [55]. Moldoveanu et al. (2022) further established that immune effector proximity to malignant cells, particularly CD8 + T lymphocytes, significantly impacts immunotherapeutic outcomes [56]. Beyond the impact of spatial distribution on therapeutic efficacy, molecular‐level analyses reveal that efficacy is associated not only with the extent of CD8^+^ T cell infiltration but also with OLR1, a gene linked to such infiltration, which itself shows a significant correlation with patient prognosis [57]. Immunoscore (IS) quantification was used to evaluate the spatial distribution of CD8 + T cells across the tumor core versus invasive margin compartments. Elevated intratumoral density (high IS) signifies robust antitumor immunity and independently predicts favorable prognosis in early‐stage NSCLC, melanoma, and colorectal malignancies [58, 59]. Elevated IS in HNSCC is inversely correlated with regulatory T‐cell infiltration but is concurrently associated with upregulated PD‐L1 and MHC class I tumor expression. These biomarker patterns demonstrate the ability of the IS to predict anti‐PD‐1/PD‐L1‐responsive disease phenotypes [60, 61].

Other T cell subtypes also significantly influence the efficacy of immunotherapy in HNSCC. A separate study revealed that early expansion of peripheral CD3^+^effector memory T cells during immunotherapy correlates with favorable treatment responses, whereas upregulation of CTLA‐4 expression on CD3^+^ T cells may portend failure of anti‐PD‐1 therapy [62]. Additionally, research focusing on IL1R2^+^Tregs demonstrated that higher abundance of this subset is associated with more advanced TNM stage and poorer prognosis, indicating its potential as a predictive biomarker for immunotherapy outcomes in HNSCC patients [63]. Regarding natural killer (NK) cells, an integrative analysis of single‐cell and spatial transcriptomic data has led to the development of a gene‐based prognostic model. This model, termed the NK & TAMs Composite Index (CINT), is founded on ligand receptor interactions between NK cells and TAMs subpopulations, specifically centering on the IL32^+^ NK‐APOE^+^ TAM axis. The CINT model has demonstrated robust performance in predicting prognosis across multiple independent cohorts [64].

#### 4.2.2 B Cell

B cell abundance has emerged as a robust predictor of immunotherapy response in HNSCC. A multimodal study integrating 10 independent cohorts including single‐cell and bulk transcriptomics, flow cytometry, and PBMC data showed that B‐cell abundance achieved a mean AUC of 0.70, outperforming CD8⁺ T cells, PD‐L1, TMB, and an IFN‐γ signature. At single‐cell resolution, the AUC rose to 0.74. Using a 5.5% cutoff in peripheral blood, an odds ratio exceeding 7.8 was consistently observed across HPV⁺ and HPV⁻ subgroups [65]. Mechanistically, B‐cell–high tumors displayed enhanced IgM expression, interferon signaling, and CXCL9 production with coordinated T/NK cell activity, whereas B‐cell–low tumors were enriched for GM‐CSF, hypoxia, and EMT programs. These findings suggest that B cells mediate antitumor immunity largely through systemic immune activation rather than tertiary lymphoid structure dependence. Given its feasibility for routine flow cytometric assessment, B‐cell abundance represents a readily translatable biomarker worthy of prospective validation. A multicenter study by N Gavrielatou et al. further demonstrated that stromal B‐cell density was the only immune cell parameter capable of significantly stratifying survival outcomes among PD‐L1 positive patients. Patients with high CD20⁺ infiltration exhibited significantly prolonged progression‐free survival, whereas those with low B‐cell infiltration responded poorly to anti–PD‐1 monotherapy despite being PD‐L1 positive, suggesting a need for combination regimens. This cohort provides the first evidence supporting “stromal B cells” as a reproducible, pathologically feasible biomarker for predicting ICI response in R/M HNSCC, and offers a rationale for intensified treatment strategies in PD‐L1–positive patients with B‐cell–deficient tumors [66]. In a separate study utilizing single cell transcriptomic data, tumor infiltrating B lymphocytes (TIL‐Bs) were classified into inhibitory and activated subsets based on their distinct gene expression profiles. Analysis with the TIDE predictor confirmed that the activated TIL‐B subset exhibited significantly lower TIDE scores and reduced T cell dysfunction scores. Furthermore, a simplified prognostic risk model based on four core genes (JCHAIN, GZMB, IGHA1, and PDRX4) was constructed, providing a potential tool for clinical translation [67]. In conclusion, these results suggest that stratifying patients by B cell functional states or their associated gene signatures holds promise as a biomarker for predicting immunotherapy response in HNSCC.

#### 4.2.3 Dendritic Cell

DCs are frequently recruited into the TME of HNSCC, where they are educated by tumor derived cytokines such as IL‐10, VEGF, and TGFβ to adopt an immunosuppressive state, thereby playing a critical role in shaping responses to immunotherapy. One study revealed that DCs in tumor draining lymph nodes (TDLNs) exhibit high PD‐L1 expression, which not only correlates with exacerbated CD8^+^ T cell exhaustion within TDLNs, but is also clinically associated with a significantly elevated risk of lymphatic metastasis or post‑operative recurrence. Consequently, PD‐L1 high cDC1s in TDLNs may serve as a robust identifier of patient subgroups that are likely to exhibit poor responses to current immunotherapies and have an unfavorable prognosis [68]. However, a recent high dimensional spatial analysis of pre‐treatment tissue samples using ultrafast cyclic immunofluorescence imaging (FAST) demonstrated that CCR7^+^ DCs located at the invasive margin of tumors express LAMP3 and PD‐L1 and support CD8^+^T cell responses to anti PD‐1 therapy via IL12 production. Moreover, perivascular CCR7^+^DCs predominantly secrete IL15, promoting T cell survival within the TME. Notably, the abundance of CCR7^+^DCs at the tumor border is significantly increased in immunotherapy responders, suggesting their potential as a predictive biomarker for treatment efficacy [69]. Similarly, work by Saito et al indicates that impaired intratumoral cDC1 infiltration may be a key determinant of resistance to anti PD‐1 therapy in HNSCC. Specifically, low cDC1 levels predict poor immunotherapy outcomes, and this deficiency is closely linked to reduced expression of the chemokine CCL5, implying that CCL5 mediates cDC1 recruitment to the tumor site. Thus, both cDC1 abundance and CCL5 expression levels hold promise as potential biomarkers for identifying HNSCC patients who may benefit from anti PD‐1 therapy [70].

#### 4.2.4 Myeloid Cell Remodeling

In HNSCC, as in various other cancers such as pancreatic, lung, and breast cancer, CAFs play critical roles in tumorigenesis, progression, metastasis, drug resistance, and immune evasion. A study by Ren et al. revealed that SPP1^+^ macrophages with stromal remodeling capacity in myeloid cells may represent a major source of antigen‐presenting CAFs (apCAFs) in HNSCC. Mechanistically, tumor cell‐secreted MIF induces the transdifferentiation of myeloid cells into CD74 high apCAFs via the JAK/STAT3 signaling pathway. The MIF‐CD74 interaction further regulates CXCL12 expression in apCAFs potentially through activation of Wnt and TGF‐β signaling. Consequently, this may facilitate the recruitment of MDSCs and native T cells, potentially leading to increased CD4^+^T cell infiltration and reduced CD8^+^ T cell presence, which may play a key role in fostering an immunosuppressive TME [71]. Furthermore, studies indicate that within the hypoxic microenvironment of HNSCC, the HIF‐α/MIF axis regulates the migration, differentiation, and pro‐angiogenic functions of CD11b^+^Gr‐1^+^ myeloid cells, thereby further promoting tumor growth. When HIF‐1α/2α is experimentally inhibited, the NF‐κB/IL‐6 axis is upregulated to compensate for the loss of HIF‐α/MIF signaling [72].

While numerous studies have focused on TME‐induced remodeling of myeloid cells and their role in promoting HNSCC progression, there remains a scarcity of myeloid cell‐based biomarkers for predicting the efficacy of related immunotherapies. Future large‐scale prospective studies are warranted to identify valuable biomarkers in this context.

#### 4.2.5 Tumor‐Specific MHC‐II

The study by Zhang et al [73]. represents the first effort to elucidate the relationship between tumor‐specific MHC class II (tsMHC‐II) expression, a hot immune TME, and response to neoadjuvant immunotherapy in HNSCC using single‐cell RNA sequencing. This work systematically evaluated the impact of tsMHC‐II on CD4⁺T cell functional states, demonstrating that tsMHC‐II not only promotes CD4⁺Th cell polarization to enhance CD8⁺cytotoxic T lymphocyte recruitment and activity but also activates a subset of CD4⁺cytotoxic T cells with independent killing capacity, thereby broadening the known functional repertoire of CD4⁺T cells in antitumor immunity. From a biomarker perspective, the study comprehensively assessed the potential of tsMHC‐II in predicting response to neoadjuvant immunotherapy in HNSCC, revealing promising predictive performance (AUC = 0.73), comparable to previously reported IFN‐γ signature (AUC = 0.75). Furthermore, the consistency between the CXCL9∶SPP1 (C/S) ratio and tsMHC‐II expression was validated, with tsMHC‐II‐high tumors exhibiting a CXCL9‐dominant, antitumoral macrophage phenotype‐supporting its role as a favorable microenvironmental marker for immunotherapy response. Although PD‐L1 CPS showed limited predictive ability in this cohort, its combination with tsMHC‐II improved predictive accuracy. In summary, tsMHC‐II emerges as an easily detectable, functionally relevant, and mechanistically grounded biomarker with promising potential for application in neoadjuvant immunotherapy strategies for HNSCC.

## 5 Circulating Biomarkers

### 5.1 Inflammatory Markers

Beyond tumor tissue‐specific biomarkers, systemic inflammatory markers in peripheral blood are increasingly recognized for their value in predicting response to immunotherapy in HNSCC. A retrospective study by Nenclares et al [74]. demonstrated that during treatment with ICI, a decrease in both the neutrophil‐to‐lymphocyte ratio (NLR) and fibrinogen levels was significantly associated with objective response and survival benefit. The predictive performance of NLR was particularly notable: using a cut‐off value of 3, it achieved a sensitivity of 0.9 for distinguishing responders from non‐responders, coupled with a high negative predictive value, making it highly suitable as a rapid clinical screening tool. Although a definitive single threshold for fibrinogen was not established, its mechanism is closely linked to IL‐6/STAT3 pathway‐driven expansion of myeloid‐derived suppressor cells (MDSCs) and upregulation of PD‐L1, indirectly reflecting a state of host systemic immunosuppression. The study further proposed integrating on‐treatment NLR and fibrinogen into an “On‐treatment Immune Prognostic Score,” which increased the area under the curve (AUC) for predicting long‐term outcomes by 10%–15% compared to any single parameter, thereby significantly enhancing prognostic discrimination.

A recent study demonstrates that a pretreatment triad of elevated soluble CD25 (sCD25), high IL‐6 levels, and a restricted T‐cell receptor (TCR) repertoire independently predicts poor prognosis in recurrent/metastatic HNSCC, with a combined hazard ratio (HR) outperforming conventional T‐stage or PD‐L1 CPS. In patients exhibiting this high‐risk profile, the addition of a JAK inhibitor (such as ruxolitinib) may simultaneously suppress tumor proliferation and MDSC function. These findings suggest that future immunotherapy strategies in R/M HNSCC should move beyond PD‐L1 CPS as a standalone biomarker, and instead adopt a multiparameter blood immunoprofile integrating IL‐6, sCD25, TCR clonality, NLR, and circulating tumor DNA (ctDNA). Such an approach could better identify patients likely to benefit from PD‐1 monotherapy, while also guiding alternative strategies such as chemotherapy or combined immunomodulatory‐immune checkpoint blockade [75].

### 5.2 TCR Clonality and Dynamics

Earlier studies have characterized the TCR repertoire in patients with locoregionally advanced HNSCC and systematically compared differences between HPV‐positive and HPV‐negative patients [76, 77]. However, these investigations failed to establish a robust correlation between TCR clonal features and treatment outcomes with ICIs. Recent research is gradually addressing this gap, highlighting the potential of TCR clonal dynamics in predicting therapeutic responses. Some investigations analyzing the TCRβ chain in PBMCs from patients with R/M HNSCC treated with cetuximab and nivolumab (anti‐PD‐1) combination therapy demonstrated that pre‐treatment TCR diversity and clonal composition were significantly associated with therapeutic response and survival outcomes [78]. These findings suggest their potential utility as predictive and prognostic biomarkers for immunotherapy. Notably, patients achieving complete response (CR) or partial response (PR) exhibited higher baseline TCR diversity, a trend more pronounced in HPV‐negative individuals or those with a smoking history [79]. A 2023 study by Ge et al. [80] provided critical insights in this field. Specifically, responders exhibited a significantly higher normalized overlap ratio of expanded high‐frequency TCR clonotypes (e.g., the top 20 or top 50 clonotypes) between PBMCs and TILs compared to non‐responders. However, this response‐associated preferential overlap was no longer detectable when the analysis included all expanded TCR clonotypes. The findings demonstrate that the expansion rate of top TCR clonotypes in peripheral blood may serve as a minimally invasive and readily accessible biomarker for predicting cetuximab response in HNSCC. Additionally, the expansion rate of top clonotypes within TILs and their overlap probability with PBMCs could provide supplementary predictive value.

### 5.3 T and B Cell Clonal Dynamics

A recent Phase II trial revealed that in the neoadjuvant immunotherapy setting for locally advanced resectable HNSCC, the transcriptional phenotypes of CD8⁺ tumor‐infiltrating lymphocytes (TILs) can effectively guide therapeutic selection. Patients achieving deep pathological response (pTR‐2) exhibited baseline enrichment of an “effector‐memory‐low inhibitory receptor” CD8⁺ T‐cell subset (characterized by low LAG‐3/HAVCR2 expression) in the Nivo^+^Ipi cohort, whereas responders in the Nivo^+^ Rela group were predominantly characterized by an “interferon‐high responsive‐pre‐exhausted” subset (with high ISG15/MX1/OAS1 expression). Mechanistically, Nivo^+^Ipi promoted clonal expansion of effector T cells, while Nivo^+^ Rela mediated tumor clearance through reactivation of a pre‐existing pre‐exhausted T‐cell population. These findings support the implementation of baseline CD8^+^ TIL immunophenotyping to guide personalized therapeutic selection in neoadjuvant immunotherapy, thereby advancing HNSCC treatment into an era of precision immuno‐oncology [8]. Building on the insights into CD8^+^ T‐cell phenotypes that guide neoadjuvant immunotherapy selection, recent investigations have further extended the scope of biomarker discovery to peripheral blood. In a study by Wang et al. [81], dynamic changes in the blood of HNSCC mouse models undergoing immune checkpoint inhibition (ICI) were systematically analyzed at multiple timepoints. The researchers identified Day 5 post‐ICB treatment as the optimal timepoint for early response assessment in mice. By integrating features of clonal expansion and gene expression in both effector memory T cells (Tem) and B cells under ICB treatment, they established a liquid biomarker signature termed LiBIO (Liquid Biomarker of Immunotherapy Outcomes). Cross‐species validation demonstrated that the LiBIO score was significantly associated with ICB response and long‐term survival in human peripheral blood and tumor transcriptomes. Moreover, LiBIO outperformed the RNA‐based consensus pathogen response signature (RNA‐CPS), indicating its potential as a non‐invasive and high‐fidelity biomarker for predicting immunotherapy efficacy in patients with HNSCC.

## 6 Novel Biomarkers From Front‐Line Therapies

### 6.1 Immune Aging Set

With the advancement of immunotherapy, the combination of neoadjuvant chemotherapy and immunotherapy has been increasingly explored in various tumors. Chemotherapy‐induced cellular senescence significantly influences the efficacy of immunotherapy [82]. Furthermore, the researchers constructed a 154 gene immune aging set (IAGs), which quantifies the degree of senescence at single‐cell resolution, addressing the limitation of peripheral blood immunophenotyping in predicting immunotherapy outcomes. The authors also emphasized that the heterogeneity of senescent cells often renders a single senolytic agent insufficient for comprehensive clearance: combinations of different agents (e.g., Navitoclax and Fisetin) exhibit complementary clearance effects across different models, though their dose–response and temporal dynamics require further optimization. Despite these challenges, the COIS‐01 trial pioneers the integration of immunotherapy with anti‐senescence strategies, offering a novel perspective and a potential biomarker framework for predicting response to immunotherapy [83]. Moreover, a study has established a predictive model based on 16 senescence‐associated lncRNAs. This signature not only effectively predicts the prognosis of patients with HNSCC but also reveals that the low‐risk group exhibits a higher potential for immune evasion and a lower likelihood of benefiting from ICB therapy. The researchers infer that the highly immune infiltrated microenvironment defined by these specific senescence‐associated lncRNAs may not simply correspond to sensitivity to ICI. Instead, it could be linked to an immunosuppressive state. In summary, further research focusing on anticellular senescence therapies is needed to elucidate their potential in remodeling the TME and predicting responses to immunotherapy [84].

### 6.2 Eftilagimod Alpha

Eftilagimod alpha (Efti), a first‐in‐class soluble LAG‐3 protein, functions by activating antigen‐presenting cells (APCs) and subsequently enhancing downstream T‐cell immunity. In the TACTI‐003 clinical trial conducted by Forster et al. [85], the combination of efti with pembrolizumab was evaluated in patients with second‐line R/M HNSCC, demonstrating substantial antitumor activity along with a favorable tolerability profile. To date, the mature median overall survival (OS) among evaluable patients reached 17.6 months, which compares favorably with historical outcomes under current standard regimens in HNSCC patients with a CPS < 1—including 10.7 months for cetuximab plus chemotherapy, 11.3 months for anti‐PD‐1 therapy combined with chemotherapy, and 7.9 months for anti‐PD‐1 monotherapy. Based on these encouraging Phase IIb data, the U.S. Food and Drug Administration (FDA) has recognized the promising activity of this combination regimen in the CPS < 1 HNSCC population and supports its continued clinical development. The study redefines the treatment landscape for CPS < 1 HNSCC patients and demonstrates substantial clinical potential. These findings also underscore the importance of exploring relevant predictive biomarkers for treatment response to precisely identify this patient subgroup and optimize therapeutic strategies.

### 6.3 Integrative Biomarkers

Relying on a single biomarker to fully predict treatment outcomes remains challenging. Given this limitation, multi‐gene models may offer superior accuracy and predictive value by enabling a more comprehensive assessment of the complexity within the TME. In one study, single‐cell and bulk RNA‐sequencing data were integrated to identify eight genes critically involved in tumor progression and immune regulation (DEFB1, AICDA, TYK2, CCR7, SCARB1, ULBP2, STC2, and LGR5). A risk model based on these genes was subsequently developed and validated. This model demonstrated that patients in the low‐risk group exhibited higher infiltration of memory activated CD4^+^ T cells, CD8^+^ T cells, and plasma cells, along with elevated immune scores. These findings suggest that compared to the high‐risk group—characterized by greater infiltration of activated mast cells and M2 macrophages—the low‐risk group is more likely to benefit from immunotherapy [86].

Although some HPV‐negative patients may achieve certain therapeutic benefits from ICIs, the overall response rate remains significantly lower than in their HPV‐positive counterparts, posing challenges for developing personalized treatment strategies for this subgroup. A recent study conducted a comprehensive proteogenomic analysis of 108 HPV‐negative HNSCC patients, integrating DNA, RNA, protein, and phosphopeptide data. This multi‐omics approach enabled the stratification of patients into three distinct molecular subtypes, with corresponding targeted therapeutic recommendations proposed for each subtype, including cyclin‐dependent kinase (CDK) inhibitors, epidermal growth factor receptor (EGFR) antibodies, and ICI therapy [87]. This research provides a new direction for precision medicine in HPV‐negative HNSCC and lays the groundwork for advancing individualized immunotherapy strategies [88]. Furthermore, HNSCC exhibits substantial intratumoral heterogeneity, which contributes to considerable variability in patient responses to conventional anti‐PD‐1/PD‐L1 immunotherapy. Recent investigations employing multi‐omics approaches to characterize molecular subtypes of HNSCC have demonstrated considerable potential for refining patient stratification. For instance, a recent study integrating miRNA, mRNA, methylation, mutation, and copy number variation data from HNSCC patients identified three distinct molecular subtypes. These subtypes exhibit significant differences in clinicopathological features, prognosis, TIME, and therapeutic susceptibility, underscoring their substantial clinical relevance [89].

## 7 Summary

In conclusion, remarkable progress has been made in immunotherapy for HNSCC, but some patients do not benefit from ICIs. Therefore, identifying biomarkers that can predict the efficacy of immunotherapy is crucial for enhancing treatment outcomes and achieving personalized therapy. Existing studies provide a theoretical basis for personalized immunotherapy, but most of these studies are retrospective and lack sufficient prospective validation. Future research should focus on the use of multiomics approaches to integrate various biomarkers, conduct more prospective clinical trials to evaluate their predictive capabilities, and analyze multiomics data with AI and machine learning. This will advance the clinical application of HNSCC immunotherapy, enabling biomarkers to predict efficacy, assess side effect risks, optimize treatment plans, and improve overall treatment effectiveness and patient quality of life. In conclusion, while immunotherapy for HNSCC shows great promise, achieving precision medicine requires more systematic and in‐depth biomarker research to guide future clinical practice and scientific inquiry.

## Author Contributions

Xiao‐Tong Yan conceived the review, curated the literature, performed formal analysis and visualization, and wrote the original and revised drafts. Zhao‐Hong An contributed to the methodology and supervised the study. Chang‐Ming An acquired funding, provided resources, supervised the project, validated the findings, and critically revised the manuscript. All authors read and approved the final manuscript.

## Ethics Statement

The authors have nothing to report.

## Conflicts of Interest

The authors declare no conflicts of interest.

## Data Availability

This article is a comprehensive review; no new, original datasets were generated or analyzed. All data discussed are publicly available from the cited peer‐reviewed literature, TCGA‐HNS.
